# The role of the healthcare sector in the prevention of sexual violence against sub-Saharan transmigrants in Morocco: a study of knowledge, attitudes and practices of healthcare workers

**DOI:** 10.1186/1472-6963-13-77

**Published:** 2013-02-25

**Authors:** Seline van den Ameele, Ines Keygnaert, Alima Rachidi, Kristien Roelens, Marleen Temmerman

**Affiliations:** 1 ICRH- International Centre for Reproductive Health, Faculty of Medicine & Health Sciences, Ghent University, De Pintelaan 185 - P3, Ghent, 9000, Belgium; 2 AFVIC – L’Association des Amis et Familles des Victimes de l’Immigration Clandestine, Casablanca, Morocco

**Keywords:** Sub-Saharan migrants, Morocco, Sexual violence, Health services, Prevention

## Abstract

**Background:**

Sub-Saharan transmigrants in Morocco are extremely vulnerable to sexual violence. From a public health perspective, the healthcare system is globally considered an important partner in the prevention of sexual violence. The aim of this study is twofold. In a first phase, we aimed to identify the current role and position of the Moroccan healthcare sector in the prevention of sexual violence against sub-Saharan transmigrants. In a second phase, we wanted these results and available guidelines to be the topic of a participatory process with local stakeholders in order to formulate recommendations for a more desirable prevention of sexual violence against sub-Saharan transmigrants by the Moroccan healthcare sector.

**Methods:**

Knowledge, attitudes and practices of healthcare workers in Morocco concerning sexual violence against sub-Saharan transmigrants and its prevention were firstly explored in semi-structured interviews after which they were discussed in a participatory process resulting in the formulation of recommendations.

**Results:**

All participants (n=24) acknowledged the need for desirable prevention of sexual violence against transmigrants. Furthermore, important barriers in tertiary prevention practices, i.e. psychosocial and judicial referral and long-term follow-up, and in secondary prevention attitudes, i.e. active identification of victims were identified. Moreover, existing services for Moroccan victims of sexual violence currently do not address the sub-Saharan population. Thus, transmigrants are bound to rely on the aid of civil society.

**Conclusions:**

This research demonstrates the low accessibility of existing Moroccan services for sub-Saharan migrants. In particular, there is an absence of prevention initiatives addressing sexual violence against the sub-Saharan transmigrant population. Although healthcare workers do wish to develop prevention initiatives, they are dealing with structural difficulties and a lack of expertise. Recommendations adapted to the context of sub-Saharan transmigrants in Morocco are suggested.

## Background

### Transmigrants in Morocco

Yearly, an estimated 65,000 to 120,000 sub-Saharan Africans cross North Africa on their way to Europe. These sub-Saharan migrants try to survive in the Maghreb countries as a region of transit, until they have saved enough to fund further migration to Europe, hence the term ‘transmigrants’. However, only one-third of these transmigrants eventually continues to Europe, as this has become increasingly difficult due to strict European immigration and Neighbourhood policies [1-3]. As a result, Northern Africa has become a region of destination rather than a region of transit. Approximately 10,000 to 15,000 sub-Saharan transmigrants are currently living in Morocco [2,4,5].

### Sexual violence as a public health problem

According to the World Health Organization (WHO), the problem of sexual violence is a public health issue [6,7]. Healthcare workers are ideally placed to recognize and intervene in sexual violence since they are frequently consulted by victims for years after the violation and are often confronted with its physical and mental health consequences.. Furthermore, healthcare workers offer long-term services, have contact with a broad range of people in both early and late stages of victimization and should offer physically and emotionally safe care in a non-judgemental and supportive way [8-10].

The healthcare sector’s response to sexual victimization can be situated in three phases of problem development: prevention of victimization; identification of persons in a violent situation and early intervention; and care for the victim after victimization (primary, secondary and tertiary prevention, respectively). In practice, healthcare’s main role concerns secondary and tertiary prevention [11-13].

Several consistent, international guidelines exist regarding the role of the healthcare sector in tertiary prevention of sexual violence. They address immediate care for victims, long-term follow-up and psychosocial and legal referral [10,13,14]. Since these guidelines do not provide an elaborated strategy for every unique context, the actual planning and implementation of sexual violence prevention and response programs relies on collaboration and partnerships with target communities, governments and national and international non-governmental organizations (NGO’s) [13].

Concerning secondary prevention, no consistent standard of care is currently globally accepted [10,15]. This can be attributed to reluctance of healthcare workers to screen their patients, to insufficient evidence on the impact of screening over the long term or to lack of effective interventions following identification [15-17]. Nevertheless, many victims of sexual violence consult healthcare workers because of masked consequences of violence (i.e. anxiety, chronic pain, irritable bowel syndrome…). Yet, because of shame or fear to be blamed, victims rarely raise the topic of sexual violence spontaneously. Hence, the undisclosed violence can result in a negative line of thought concerning the experience with increasing isolation, feelings of guilt, shame and worthlessness. Without any intervention, it is assumed that symptoms persist, aggravate and that the risk of revictimization increases [10,11,18].

Research on women’s perceptions of being asked about sexual violence revealed that most women find it appropriate to be asked about it by healthcare workers as the latter are regarded as reliable and having knowledge on adequate care and referral possibilities. Women find it easier if the healthcare worker opens up the dialogue and empowers them to talk about their experiences. Risk of stigmatizing or offending should be reduced by ensuring a respectful attitude and explaining the reasons for asking about sexual violence [10,19,20].

Thus, secondary prevention, performed in a sensitive, non-judgmental way could be a first step to help victims of sexual violence to relieve themselves of their undisclosed experiences and to offer alternatives to feelings of shame and guilt. In this respect, knowledge and awareness of healthcare workers about these processes seems essential [10,19,20].

### Sexual violence in Morocco – situation of sub-Saharan transmigrants

In the last decennia, awareness on sexual violence against women and children in Morocco has substantially raised. In 2002, growing attention resulted in the ‘National Strategy for the Elimination of Violence Against Women’. This multi-sectorial strategy had been developed in a collaborative approach between government and NGO’s. It encompasses a yearly awareness-raising campaign, an expanding number of psychological and legal counseling centers for victims of sexual violence and the creation of reception centers in courts and medical centers. These projects, initiated by the government, are focusing on female Moroccan victims solely and are primarily carried out by specialized NGO’s [21,22].

Sub-Saharan transmigrants in Morocco face serious violations of their reproductive and sexual rights. Several reports ascribe the extreme vulnerability of the sub-Saharan transmigrants to their poor living conditions and irregular situation. They denounce the high incidence of rape of sub-Saharan transmigrants committed by gangs and soldiers in the Algerian–Moroccan border region (“no man’s land”), of trafficking and of forced prostitution of sub-Saharan women [3,4,23,24]. From April 2003 to May 2005, Médecins Sans Frontières (MSF) dealt with complaints related to violence in 25% of their medical consultations with sub-Saharan transmigrants. In 65% of the cases, Moroccan or Spanish police forces committed the violence; gangs and human traffickers were responsible for the other 35% [23]. It needs to be stressed that in transmigrants, women, as well as men and children are victimized [3,4,23,24]. At time of publication, severe violent incidents against sub-Saharan transmigrants are still being reported [25,26]. Furthermore transmigrants face several difficulties in order to access healthcare services. It ranges from fear of authorities, expulsion and stigmatization to other financial, linguistic and cultural barriers that hamper transmigrants to consult public health services [4,23].

The Moroccan healthcare system is a mixed public-private system with a compulsory insurance system guaranteeing access to public healthcare services for Moroccan citizens [27]. Current healthcare services for migrants in Morocco are essentially organized by civil society. Several NGO’s offer medical care to transmigrants and propagate their safe access to free public health services [4,23,24,28].

Growing awareness among United Nations specialized agencies, NGO’s and researchers on reproductive healthcare has given rise to numerous recommendations to promote sexual and reproductive health among refugees and asylum-seekers [13,29,30]. However, existing recommendations are not tailored to the context of sub-Saharan transmigrants in Morocco. Therefore, this research project on the role of the healthcare system regarding the problem of sexual violence and sub-Saharan transmigrants in Morocco was set up.

### Research questions

In order to identify the current role and position of the healthcare sector in Morocco in the prevention of sexual violence against sub-Saharan transmigrants, we questioned Moroccan healthcare workers on their knowledge, attitudes and practices concerning this particular problem within the three stages of prevention. We also inquired about their perceived needs to provide desirable prevention of sexual violence against sub-Saharan transmigrants. The results were taken as a starting point for a participatory debate with local stakeholders resulting in the formulation of recommendations concerning the desirable future response of the healthcare sector in Morocco to sexual violence against sub-Saharan migrants.

## Methods

### Conceptual framework: a socio-ecological perspective on health and desirable prevention

The existence of a complex health problem such as sexual violence can be explained by means of the socio-ecological model. This approach assumes that violence is the consequence of a permanent dynamic interaction between determinants situated on four levels: individual, relational, community and society [31]. Preventive interventions are most effective when they are multidisciplinary in nature, when they aim simultaneously and concordantly at the four levels of the socio-ecological model and, as mentioned earlier, when they target all stages of problem development, i.e. primary, secondary and tertiary prevention [14,32-34].

To evaluate the effectiveness of prevention initiatives, the criteria of ‘desirable prevention’ are used. Whereas general prevention can be conceived of in terms of those initiatives which anticipate risk factors in a targeted and systematic way, ‘desirable prevention’ can be defined as ‘those initiatives which anticipate risk factors ever earlier in a targeted and systematic way, are maximally “offensive”, have an integral approach, work in a participatory way and have a democratic nature, while aiming at the enhancement or protection of the target group’s health and wellbeing’ [35]. The recommendations issued from this research project are embedded in this conceptual framework.

### Study context: a participatory partnership

This study is part of a larger Belgian-Moroccan research project. The goals of the larger project are: to identify decisive determinants of sexual violence against transmigrants, to explore the experiences of transmigrants with sexual violence, and to formulate potential prevention activities. Applying a ‘Community Based Participatory Research (CBPR)’ method, this project was conducted in a close participatory partnership between Belgian and Moroccan researchers and an extensive Moroccan Community Advisory Board (CAB). The CAB consisted of transmigrants, NGO’s and other key-actors working with sub-Saharan transmigrants and working on sexual health in Morocco. Aspiring sustainable change, participation is emphasized in every step of the project.

In line with this participatory framework, our results and interpretations were scrupulously verified by the CAB and other local actors and transmigrants at the seminar ‘Sexual Violence and Sub-Saharan Transmigrants in Morocco’ (University of Mohammed V, Rabat, May 6th 2009).

### Study design

A KAP (Knowledge, Attitudes, Practices) questionnaire, identifying knowledge, attitudes and practices, guided the semi-structured interviews. The underlying theory is that information has to change knowledge and attitudes successively in order to affect healthcare workers’ behaviour and practices. A KAP study aims to identify potential barriers to behaviour change on each of these three levels [36-38].

The questionnaire consisted of 44 questions, including 8 open questions. Closed questions, with answers scored by a five- or six-point Likert scale, scrutinized healthcare workers’ knowledge, attitudes and practices regarding sexual violence. The content of these questions was based on United Nations High Commissioner of Refugees (UNHCR) guidelines concerning the response of the healthcare sector to sexual violence against refugees and asylum-seekers [13], and the guidelines of the United Nations Population Fund (UNFPA) on the identification of hidden victims of sexual violence [10]. The closed design was extended by the possibility to comment on the topic of the questions. The open questions assessed potential differences in dealing with sub-Saharan or Moroccan patients, as well as specific needs of healthcare workers who are working with sub-Saharan transmigrants in Morocco. Although the format of the questionnaire allowed written participation, most participating healthcare workers preferred a personal interview, enabling them to expound their point of view on both open and closed questions. To ensure the feasibility, completeness and relevance of the questionnaire in the Moroccan context, a Moroccan professor in sociology, a Congolese and a Moroccan physician, and a Congolese refugee-nurse piloted the questionnaire. The piloting resulted in a few linguistic changes. A Belgian female medical student conducted all interviews in French. The interviewer took notes, and most interviews were recorded. The Ethics Committee of the Ghent University Hospital approved the study.

### Recruitment

Given the indications of our CAB and literature review we initially opted for recruitment in Rabat, Oujda, Casablanca and Tangier as they are sheltering the largest number of residing sub-Saharan transmigrants. However, due to emergency security reasons at the time of the field research, most transmigrants tried to flee from Oujda to Fes. Subsequently, we were forced to replace Oujda by Fes. This resulted in recruiting participants in Rabat, Fes, Casablanca and Tangiers between June 20 and July 30, 2008.

A snowball sampling strategy was used. Initially, our sampling goal was to include first-line healthcare workers (general practitioners, community workers, obstetricians) in Morocco who are confronted with both the Moroccan and the sub-Saharan population. However, in the field, these inclusion criteria appeared too vigorous and unrealistic. Therefore, recruitment was refocused on key-persons and key-organizations specialized in either sexual violence in general in Morocco, or in healthcare for sub-Saharan transmigrants in Morocco. First-wave participants were healthcare workers either suggested by members of the CAB or found by online searches. Subsequently, these first-wave participants, in turn, indicated other potential participants. During recruitment we were confronted with saturation of the sample. Participants could only indicate healthcare workers who were already recruited. At the end of the period of field research, however, some new actors turned up.

Eligible healthcare workers were first contacted by email, later by telephone or by a personal meeting in the field. Each participant signed the informed consent, except one who participated through email. Anonymous participation was possible, but most responders provided their contact details.

### Data analysis

Interviews were transcribed *ad verbatim*. NVivo8 was used for analysis because of the experience of the researchers. Transcripts and field notes were closely read and scrupulously coded and organized in a tree structure. This structure was systematically reviewed and refined during analysis [39]. The master’s student who carried out the interviews did the coding and analysis. A researcher experienced in the field of qualitative and participatory research supervised the whole process.

To ensure reliability of our research, the criteria of Lincoln and Guba were applied. These criteria of credibility, transferability, dependability and confirmability were guaranteed by means of a) triangulation of resources (formal and informal interviews, observations, literature, media etc.); b) verification of results by participants and experts; c) a diverse profile of participants; d) participation of healthcare workers and transmigrants during the whole research project; e) diverse background of researchers; f) thorough description of results, preparation work, activities on the ground, recruitment and analysis and accurate coding; and finally g) extensive reflection on difficulties and limitations [40,41].

## Results

### Results of recruitment

Of the 57 targeted healthcare workers or organizations, 24 people agreed to participate and 2 contacts agreed to an informal conversation on the subject. The results of those 2 interviews were not included in the formal analysis, but provided important background information. There were 7 non-responders and 24 refusals. Reasons for refusal included “no experience with sexual violence or with sub-Saharan patients”(n:10); “holiday”(n:5); “lack of time”(n:4); “fear of damaging the relationship of trust with the transmigrant”(n:3); “bad experiences with previous research”(n:1) and “no formal permission from the ministry to engage in research”(n:1). Refusal to participate predominantly came from the public healthcare sector, mostly because of lack of experience with sexual violence or sub-Saharan patients. In Rabat, the positive response rate of the targeted NGO’s active in the field of transmigrant health, mounted to 75%. Both national and international NGO’s participated.

### Profile of participants

Within the group of 24 participants, there were 12 men and 12 women from different age groups. Ten of the participants were originally from Morocco, 12 from several sub-Saharan countries, 2 from France and 1 from Argentina. Three out of four participants worked in Rabat, 4 in Casablanca and 4 in Fes. Some of the participants worked in multiple Moroccan cities. One participant, active in Médecins du Monde (MDM), was operating in both France and Rabat. Participants were predominantly medical doctors and community health workers. For more details on the profile of participants see Table 1. Most participants (n=14) were employed by NGO’s. Seven other participants were employed by both NGO’s and the public health sector, while 3 were exclusively employed by the public health sector (Table 2).

**Table 1:** Profile of participants (N=24)

	**N**		**N**
*Sex*		*Religion*	
Male	12	Muslim	11
Female	12	Christian	6
*Age*		Other	2
18-29	6	Unknown	5
30-49	13	*Working area*	
50-59	5	Rabat	18
*Country of origin*		Casablanca	4
Morocco	10	Fes	4
DRC	6	Oujda	1
Burkina-Faso	1	Tangiers	1
Togo	1	France	1
Sierra Leone	1	*Function*	
Ivory Coast	1	Medical doctor	8
Senegal	1	Community health worker	7
France	2	Relief worker reception centre	2
Argentina	1	Coordinator (MDM/UNAIDS)	2
*Highest level of education*		Nurse	2
Secondary	2	Midwife	1
Higher, non-university	8	Medical student in practice	1
University	14	Psychometrician	1

**Table 2:** Origin of patient group in relation to participants’ employment (N=24)

	***Origin patient group:***			
	**Sub-Saharan**	**Moroccan/sub-Saharan**	**Moroccan**	**French/sub-Saharan**
*Participant employed in:*				
NGO	6	5	2	1
NGO & public health sector	0	7	0	0
Public health sector	0	1	2	0

### Knowledge on prevalence and determinants of sexual violence

Closed questions with a five-point Likert scale assessed the estimated prevalence of sexual violence in Morocco against women, children and men respectively and assessed the estimated frequency of confrontation of healthcare workers with victims of sexual violence. Results of these questions are found in Table 3.

**Table 3:** Estimated frequency of sexual violence in Morocco by healthcare workers and their confrontation with victims (N=24)

	**Very frequently**	**Frequently**	**Sometimes**	**Rarely**	**Never**	**I don’t know**
*Estimated frequency of sexual violence in Morocco in:*						
women	4	14	6	0	0	0
children	2	7	10	2	0	3
men	0	3	8	7	2	4
*Confrontation with victims of sexual violence*	2	3	5	5	3	1

All participants agreed on the existence of sexual victimization of women in Morocco. Likewise, there was a general agreement on the occurrence of sexual victimization of children, with only 3 participants being uncertain on this matter. Eighteen participants reported that men are sexually victimized while 4 were uncertain and 2 indicated that this phenomenon did not exist. Five participants reported frequent confrontation with victims of sexual violence. All participants acknowledged the general vulnerability of transmigrants to sexual violence. However, most of them feel unable to compare the vulnerability of Moroccan people to that of sub-Saharan transmigrants. At the same time, they emphasize the general occurrence of sexual violence within the Moroccan society.

**Figure 1:**
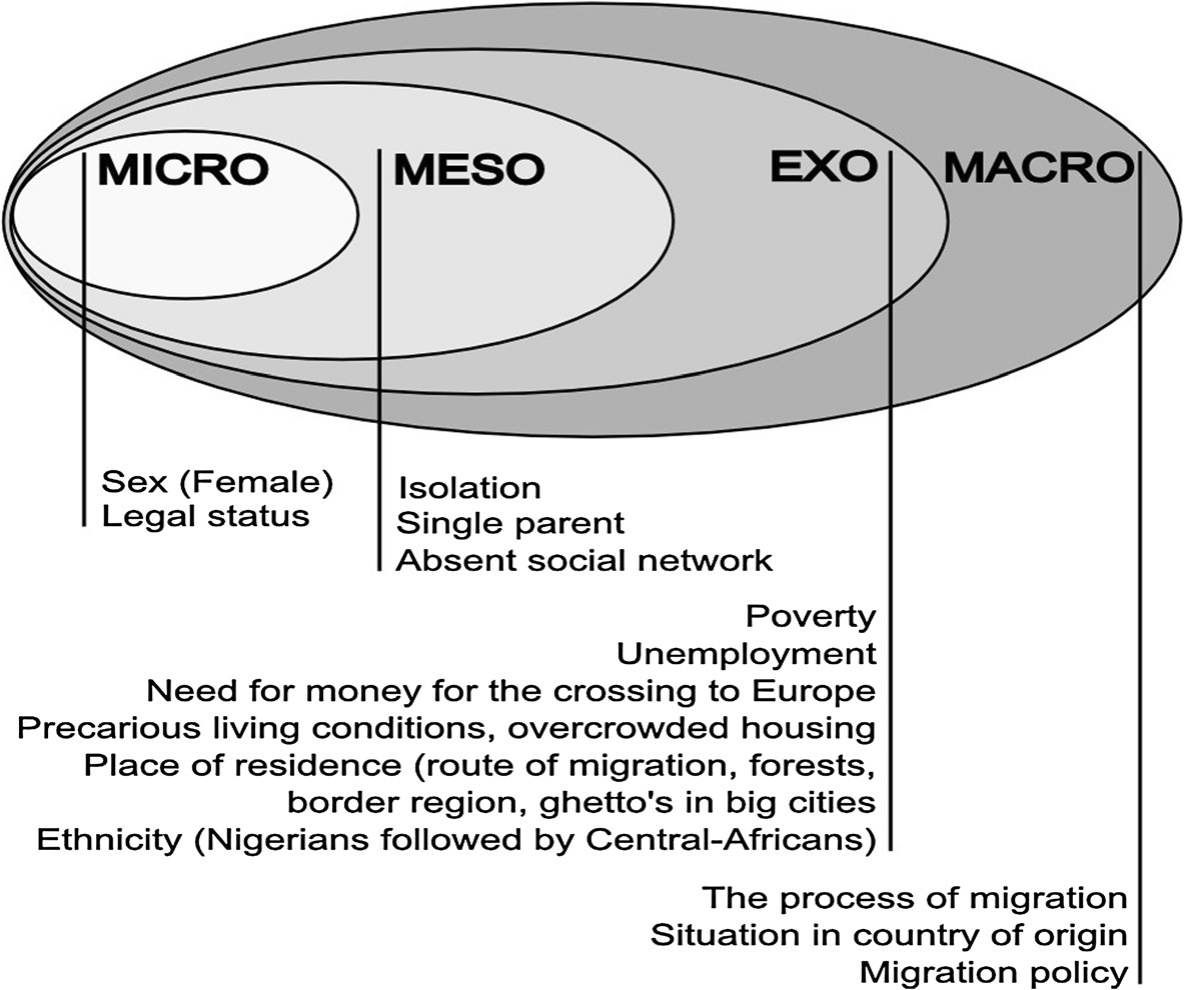
Determinants of sexual violence against sub-Saharan transmigrants in Morocco specified by healthcare workers.

The participating healthcare workers raised several determinants of sexual violence against sub-Saharan transmigrants in Morocco. Female sex, legal status, precarious living condition and ethnicity were mentioned most frequently. The determinants were organized in accordance with the socio-ecological model in micro-, meso-, exo- and macrolevel respectively in Figure 1.

Following quote illustrates the high incidence of sexual violence against sub-Saharan transmigrants in the region of Oujda:

« In the forest of Oujda, when you have the bad luck to meet them, they rape you, they take everything, your money, everything. (…) Men and women are raped in Oujda. In Oujda, there are a lot of killings. Over there they rape you, they kill you, they rape you, they kill you.»

- Physician and community health worker, Democratic Republic of Congo (DRC)

### Attitudes and practices on the level of tertiary prevention

Based on the UNHCR guidelines [13], attitudes and practices concerning care for injuries, pregnancy testing, sexual transmittable infections (STI)-screening and treatment, abortion, psychosocial and legal assistance and long-term follow-up were questioned.

Attitudes concerning the practice of immediate care after victimization, i.e. care for injuries, pregnancy testing and STI-screening and treatment, largely matched the UNHCR guidelines. However on the subject of abortion, there was great reticence to make a statement. There was unanimous confirmation of the importance of psychological assistance. Most participants agreed that healthcare workers should assist in guiding patients towards a safer living environment, although they also emphasized the difficulties related to it. Likewise, these participants considered it their responsibility to provide evidence of the violence and to refer victims to legal assistance. In the interviews several objections were raised: ‘legal issues are not within the scope of healthcare’, ‘it is not possible to document violence that took place during the route of migration’, ‘reporting will not help the victim’, ‘the police reacts only in cases of Moroccan victims’, ‘illegal transmigrants risk being deported’, ‘healthcare workers are unaware of existing legal procedures’. Finally, a small majority of participants agreed on the need for long-term follow-up regarding physical and reproductive health. Some participants were skeptical and mentioned difficulties, such as the intrinsic mobility of transmigrants and the low accessibility of certain communities. They limit the healthcare worker’s responsibility of long-term follow-up to one year after victimization and outsource later follow-up to psychologists.

Practices concerning immediate care for victims of sexual violence are in line with the above mentioned attitudes. For religious and legal reasons, few healthcare workers carry out abortions. Most participants reported referring victims to psychological help. Half of them stated that they try to guide victims towards a safer living environment. The participating healthcare workers did not collect evidence of violence, which was justified by ‘difficulties in Morocco’, ‘lack of time’ or ‘someone else’s responsibility’. Half of the participants reported organizing judicial referral. In practice, long-term follow-up did not take place.

### Attitudes and practices on the level of secondary prevention

Attitudes on whether healthcare workers have the responsibility to actively identify victims are presented in Table 4. Half of the participants argue that the active identification of hidden victims is one of the responsibilities of healthcare workers. The other half does not report an opinion or does not believe identification belongs to their professional duty:

“No, I don’t think a healthcare worker should look for health problems. We feel more comfortable being consulted than when we provoke problems. Otherwise we will find sick persons everywhere.” (Physician, DRC)

**Table 4:** Attitudes of participants towards active identification of hidden victims by healthcare workers

**Attitudes**	**Number of participants (N=24)**
*Pro*	*12*
Healthcare workers are in a position of confidence	5
Identification is crucial	5
Healthcare workers are responsible for diagnosing accurately and treating properly	1
Medical attestation is required for legal procedures	1
*Contra*	*8*
Not a specific responsibility of the healthcare worker	3
Active searching for victims is not done	3
Risk of raising bad ideas in society	1
Lack of time	1
*No opinion*	*4*

Three out of 4 participants agreed that making enquiries with the aim of identifying potential victims could be a first step to ending the violence and its consequences. Some expounded this point of view, stating that ending the violence is too ambitious; indicating that contributing to the reduction of violence is more realistic.

Regarding practices, ten participants claimed to systematically ask their patients about sexual violence when they suspect them to be victims. Nine participants avoid asking about it for various reasons. Indicated barriers are listed below. These ranged from not feeling competent or not having the right intervention possibilities, to believing that enquiring about violence is not in the patients’ best interest. Others approach the issue in an indirect way and encourage victims to bring up the topic themselves. Some said that they need obvious evidence of sexual violence before mentioning it.

Barriers to questioning patients about possible sexual violence when suspecting it

–Not among the competences of healthcare workers

–No appropriate possibilities to intervene after identification of a victim

–Anxiety of offending the victim

–Identification is not part of the mandate of some healthcare workers

–Lack of time

–Reluctance of healthcare worker to open up old wounds

–Respect for the victim’s choice to maintain silence

“The victim has to take her own responsibility; when she wants help, she will confide her problem. (…) Unfortunately, I don’t know how to deal with this issue. I don’t know who to refer to.” (Physician and community health worker, DRC)

“The community health workers meet the people, they have to enter upon the subject of rape. I don’t do it because it is not part of my project. Community health workers who enquire about sexual violence do not exist.” (Community health worker, DRC)

### Role of the public health sector versus NGO’s in the prevention of sexual violence

The participants’ overall attitude is that the public health sector should play a leading role in the prevention of sexual violence and this within the three stages of prevention. In practice, several participants indicated that transmigrants rely entirely on help from NGO’s. There are cultural, legal and financial limitations to the current response of the public healthcare sector for both Moroccan and sub-Saharan victims of sexual violence. The limitations of the Moroccan public health sector regarding the response to sexual violence mentioned by the participating healthcare workers are listed below.

Limitations of the Moroccan public health sector regarding the response to sexual violence

General limitations

–Medical sector provides primarily physical care after violence

–Violence in women is hushed up in Moroccan society

–Insufficient staffing, structures and resources

–Insufficient awareness among healthcare workers

Specific limitations for transmigrants

–Differences in culture, language and religion

–Lack of financial means

–Anxiety of being expelled

–Reticence of medical staff towards transmigrants

–Misperception of sexual liberty in sub-Saharan culture

### Needs of the healthcare sector regarding the response to sexual violence

A great need for education was reported. Participants described the need for competent and sensitized staff. They wished to gain better insight into the current possibilities of assistance (medical, psychological and legal) for both Moroccan and sub-Saharan victims of sexual violence. Almost all participants emphasized the importance of creating a specialized, multidisciplinary structure that engages in an overall approach to sexual violence and that collaborate in a more coordinated and structured way. They also stressed the importance of formal inclusion of sub-Saharan transmigrants in the ‘National Strategy for the Elimination of Violence Against Women’, with special attention to the current migration policy and accessibility to healthcare.

## Discussion

Our results demonstrate that all participants recognize the need for desirable prevention of sexual violence against transmigrants. We identified important barriers in tertiary prevention practices, i.e. psychosocial and judicial referral and long-term follow-up, and in secondary prevention attitudes, i.e. active identification of victims. Existing services for Moroccan victims of sexual violence do not yet address the sub-Saharan population. Thus, transmigrants are bound to rely on the aid of the civil society.

### Discussion of methods

Initially, our sampling aim was to include first-line healthcare workers confronted with both the Moroccan as well as the sub-Saharan population. However, in the field, these inclusion criteria appeared to be too vigorous and unrealistic, as there seem to exist two parallel circuits in which respectively sub-Saharan healthcare workers see sub-Saharan patients, and Moroccan healthcare workers see predominantly native Moroccan patients. Only a few Moroccan healthcare workers active within the public sector also work in an NGO and have experience with both populations. Therefore, recruitment was refocused on key-persons and key-organizations specialized in either sexual violence in general in Morocco, or in healthcare for sub-Saharan transmigrants in Morocco.

Despite extensive recruitment efforts in all 4 cities, only one fourth of the responding healthcare workers that were included, are operating outside of Rabat. This reflects a relatively higher density of services for sub-Saharan transmigrants in the capital.

For Moroccan patients as well as for transmigrants, the organization of the Moroccan network engaged in reproductive and sexual health lacks transparency and structure. This lack hampered the search for participants and resulted in a false early saturation of sampling. At the end of the period of field research, however, some new actors turned up. The high rate of non-responders and of refusals from the public healthcare sector, due to a reported lack of experience with sub-Saharan patients or with sexual violence in general, consequently results in an underrepresentation of the public healthcare sector. This can be seen as important information, as this might reflect lack of knowledge, taboo, anxiety and low accessibility, especially bearing in mind the high prevalence of sexual violence in Morocco [21].

The areas of field research, i.e. Rabat, Casablanca, Tanger and Fes, were contingent on the larger Belgian-Moroccan research project and they aimed to cover areas in Morocco sheltering the largest number of residing sub-Saharan transmigrants. Several participants reported a high incidence of sexual violence in Oujda and in the Algerian border region. As there is only one participant with working-experience in Oujda, this might have biased the results and underestimated the urgency of a response to sexual violence in this area.

Using a KAP-questionnaire to identify current practices forces researchers to rely on the veracity of the answers of the participants. Awareness of the importance of some practices could influence participants towards desirable answering. However, we believe that the diverse profile of participants and critical analysis of results together with transmigrants and healthcare workers, helped to diminish the impact of this possible bias when drawing conclusions on current practices.

The criteria of Lincoln and Guba were applied to ensure reliability of our research. Confirmability, dependability and credibility in particular were strengthened by participation of healthcare workers and transmigrants during the whole research project and by verification of the results by experts at a local seminar. Difficulties in recruitment and underrepresentation of the public healthcare sector might however reduce credibility of our research [40,41].

### Prevalence and determinants of sexual violence

The responding healthcare workers were aware of the existence of sexual violence against women and children and of the particular vulnerability of transmigrants. Simultaneous research of Keygnaert et al. [42] confirms the high incidence of sexual violence, 89% of 154 interviewed transmigrants reported a total of 548 acts of violence in their close environment. These acts involved sexual violence in 45% of the cases. Notwithstanding the fact that transmigrants in the research of Keygnaert et al. [42] reported male victims in 36% of the reported cases of rape, most health workers interviewed in this study are not convinced of the occurrence of male victimization.

Despite the estimated high frequency of sexual violence, the responding health workers indicated that they rarely came into contact with victims. This discrepancy makes an underestimation by healthcare workers of actual contact with victims of sexual violence very probable.

Determinants of sexual violence indicated by our participants were similar to those reported by the interviewed transmigrants in the research of Keygnaert et al. [42]. Although the vulnerability of Moroccan people is emphasized, participants were aware of an extreme vulnerability of sub-Saharan transmigrants, and this on all levels of the socio-ecological model.

### Tertiary prevention

Attitudes of healthcare workers in their response to sexual violence roughly match the suggested approach of the UNHCR guidelines [13]. These attitudes are put into practice without any adaptation to the local context nor standardization. There is a lack of knowledge and there are structural difficulties in long-term follow-up, psychosocial and judicial referral, which has repercussions on current practices. Awareness of the importance of psychosocial support might bias the response of participants towards desirable behaviour when reporting their practices. The numerous obstacles that were revealed suggest a discrepancy between reported and actual practices.

The lack of transparent procedures and the presence of structural and legal difficulties account for a discrepancy between attitudes and practices within the legal aspects of the healthcare response to sexual violence.

There hardly is any long-term follow-up, as it is hindered by the attitude that the responsibility of the physician ends when physical problems are healed. Physical long term-consequences of sexual violence were not taken into account and follow-up was believed to be the domain of psychologists. The intrinsic mobility of the transmigrant population might complicate actual follow-up.

### Secondary prevention

Most participants do not recognize the usefulness of active victim identification practices. Several participants indicated that systematic screening of potential victims would not help to reduce sexual violence. They motivated this point of view by stating that the highest prevalence of violence was found at the border. They deem care unnecessary for victims of violence that has already taken place during the route of migration. This illustrates a lack of knowledge concerning the utility of secondary prevention.

It is difficult to draw conclusions from the responses of the 10 participants stating that they systematically broach the topic of sexual violence when suspecting it. Questions related to the signs of violence demonstrated that the healthcare workers were mainly alerted to immediate consequences of sexual violence.

The results suggest that in current practice, unless there are obvious signs of sexual violence, the majority of healthcare workers prefers to adopt an open, but passive attitude. Most participants were motivated to identify victims of sexual violence, but their current lack of competences and of appropriate interventions makes the healthcare workers to wait for the victims’ initiative.

The barriers at the levels of knowledge, attitudes and practices, which were identified in this study, are similar to those identified in research on screening for intimate partner violence by healthcare workers [11,15,16,19,37]. Bearing in mind current opinion on women’s perceptions of being asked about sexual violence, a first challenge on this prevention level seems to be awareness-raising and adequate training of healthcare workers in order to help them understand the behaviour and needs of victims of sexual violence [19,20].

### Role of the public healthcare sector versus NGO’s

The gap between the public healthcare sector and NGO’s is striking in the Moroccan healthcare system. All participants, with the exception of participants working exclusively in the public health sector, indicated that healthcare for transmigrants lies entirely in the hands of NGO’s. Although transmigrants’ access to the public health sector is possibly in theory, many barriers were mentioned. These findings confirm the reported low accessibility of Moroccan public healthcare for migrants in earlier research [4,23,24].

It is unclear to what extent the participants’ perceptions are influenced by the fact that they are familiar with either NGO’s or public services. The limited number of healthcare workers working exclusively in the public sector does not allow their opinions to be compared to those working in NGO’s. Nevertheless, the opinions of participants working in both settings largely correspond to those working exclusively in NGO’s.

In Rabat and Casablanca we came upon many valuable initiatives promoting the integration of transmigrants into the Moroccan healthcare system. Most of these initiatives were organized by and in collaboration with the Moroccan government. We consider the improvement of governmental support for these initiatives as essential.

### Needs of the healthcare sector regarding sexual violence

ur interviews revealed a current absence of prevention measures, as well as an unequivocal wish for prevention initiatives. The most prominent need reported is adequate training of healthcare workers. Many participants working only with transmigrants seem uninformed about existing initiatives for Moroccan victims of sexual violence. Hence these existing initiatives are not optimally being used for transmigrants. However, the assistance for Moroccan victims is not problem-free either. These projects for Moroccan victims only started recently and need further development.

## Recommendations

Our proposed recommendations encompass actions taken by healthcare workers within the three stages of prevention and on the micro, meso, exo and macro levels of the socio-ecological model. These recommendations were developed based on the results of the KAP study and inspired by the UNHCR [13] and UNFPA [10] guidelines. They have been scrupulously reviewed and modified by local actors and transmigrants at the seminar ‘Sexual Violence and Sub-Saharan Transmigrants in Morocco’ (University of Mohammed V, Rabat, May 6th 2009). Because of the active participation of the target population and due to the application of the socio-ecological concept of violence and the three-tier prevention model during the whole research project, the proposed recommendations fulfil the criteria of desirable prevention to a great extent.

### Tertiary prevention

Provide accessible, immediate and reliable care that inspires confidence

–Provide the ability for direct registration, or by way of a community health worker, in an accessible first-line health centre or reception centre for sexual violence. Communicate the confidentiality to the sub-Saharan community.

–A standardized protocol should encompass all aspects of immediate care and long-term follow-up.

### Secondary prevention

Aim at early identification of victims of sexual violence

–Be aware of potential barriers (such as shame, anxiety, culture, language) that hinder victims from speaking spontaneously about their experience of violence.

–Gain insight into personal barriers to dealing with sexual violence.

–Provide an immediate and appropriate response after the identification of victims.

–Avoid stigmatization of the sub-Saharan population in the field of sexual violence.

–Incorporate the identification of hidden victims of sexual violence in community health projects.

### Primary prevention

Prevention is better than cure

–Operate with community health workers to disseminate messages of prevention and protection.

–Exert pressure on the government by reporting data.

–Identify factors that contribute to vulnerability, and adopt prevention campaigns.

–Organize activities in the field of reproductive health.Awareness-raising and information campaigns on the topic of HIV/AIDS could be extended with activities and discussions in the sphere of gender, sexuality and gender-based violence. Men also have to be involved in these activities.

### All stages of prevention

Create a specialized and multidisciplinary structure/network for an inclusive approach to sexual violence

–Collaborate with staff operating in the field of health, protection, education, psychology, law, community work etc.

–Form networks consisting of the target population, community health workers, reception centers, health centers, the police and specialized organizations.

–Optimize the activity and the number of existing reception centers for victims of sexual violence.

–Include transmigrants in the national strategy for combating sexual violence against women.

–Identify Moroccan organizations engaged in the struggle against sexual violence that are willing to include transmigrants in their activities. Collaborate with organizations that have transmigrants as a target population and currently work principally on HIV/AIDS.

Integrate sub-Saharan transmigrants into the Moroccan public health sector

–Train healthcare workers to be competent to deal with transmigrants: awareness of the influence of culture and origin on health; knowledge of the possibility of (sexual) traumatization during migration and the impact of this over the long term; knowledge of the context of living in Morocco; awareness of the unstable character of a transmigrant population.

–Organize an approach that takes linguistic, social, economic and administrative problems into account.

–Confer with the Ministry of Health and NGOs on mediation between the target group and hospitals/health centers.

–Formalize access to care in the form of an official report by the Ministry of Health.

–Analyze other barriers to care for transmigrants and eliminate them.

Operate with adequate trained, specialized, diverse and sufficient staff

–All staff coming into contact with potential victims of sexual violence need to be informed about themes such as gender, sexual violence, migration, sexuality and cultural differences. Sympathy and respect are essential in dealing with sexuality and reproductive health.

–Diversify staff regarding to language, origin, religion, gender etc.

–Develop and organize an adequate training for all healthcare workers.

–Specific capacities:

Community health workers:

–are recruited out of different sub-Saharan communities;

–speak the language of the target population;

have a mutual relationship of trust with the target population;

–are competent at applying a screening tool;

–are able to offer first-time psychosocial support;

–have knowledge of procedures of reporting and referral to appropriate centres; and

–participate in awareness-raising.

Physicians/Nurses:

–know the protocol of the immediate approach to sexual violence;

–are aware of the long-term consequences of sexual violence;

–are aware of the importance and the urgency of HIV prophylaxis;

–have knowledge of procedures of follow-up and further referral;

–are competent at applying a screening tool; and

–follow a specific code of conduct, with respect for anonymity.

### Conclusions

Violence against women is a rising subject of attention in Morocco. Numerous initiatives for prevention and response are starting up. In contrary to the inclusive approach of a desirable prevention, current prevention initiatives have limited accessibility for sub-Saharan transmigrants. At the same time, transmigrants, NGO’s and healthcare workers engaged with transmigrants express a strong wish to develop desirable prevention of sexual violence against transmigrants. All agree that, in order to set up sustainable and effective interventions, a framework and/or initiator is required. Phenomena like these could become the foundations of a productive collaboration between the current Moroccan policy on sexual violence, the population of transmigrants and the healthcare workers. Taking into account the aspects discussed in above recommendations, this collaboration should evolve into a multidisciplinary approach considering the global context of a transmigrant in Morocco.

This research provides an impression of the knowledge, attitudes and practices of healthcare workers experienced in the field of sexual violence and/or sub-Saharan transmigrants in Morocco. We are convinced that it can serve as a leverage for further research on more specific topics such as screening-practices in Moroccan healthcare, cultural difficulties for Moroccan healthcare workers confronted with sub-Saharan patients, methods of awareness-raising, and, development and efficacy of training programs. However, the results of this research can already be considered as a call for the urgent creation and evaluation of pilot interventions as proposed in the above recommendations.

Discussing prevention in general remains incomplete without reflection on primary prevention. Primary prevention of sexual violence against transmigrants needs drastic changes in politics, economics, ethics and migration policies of African and European countries. The authors are convinced that, before developing and setting up secondary and tertiary prevention initiatives, the feasibility of primary prevention initiatives should be considered, as they are the basic condition for sustainable and effective change.

## Abbreviations

CAB: Community advisory board; DRC: Democratic Republic of Congo; KAP: Knowledge, attitudes, practices; MDM: Médecins du monde; NGO: Non-governmental organization; STI: Sexual transmittable infection; UNFPA: United Nations Population Fund; UNHCR: United Nations High Commissioner of Refugees; WHO: World Health Organization

## Competing interests

The study is part of a larger research project funded by the National Lottery of Belgium. The authors assure the absence of competing interests.

A preliminary version of this paper was presented at the project seminar ‘Sexual Violence and Sub-Saharan Transmigrants in Morocco’ in Rabat in May 2009. Research findings are extensively described in the Master’s dissertation ‘Role and position of the healthcare sector in the prevention of sexual violence against sub-Saharan transmigrants in Morocco’ by SVDA.

## Authors’ contributions

SVDA contributed to the study design, acquisition of data, analysis and manuscript writing. IK supervised the study process from design to manuscript editing. IK, KR and MT critically reviewed the manuscript for intellectual content. AR participated in study design and acquisition of data. All authors read and approved the final manuscript.

## Pre-publication history

The pre-publication history for this paper can be accessed here:

http://www.biomedcentral.com/1472-6963/13/77/prepub
